# One Health: Action in Brazilian Cases of Sporotrichosis in Humans and Cats

**DOI:** 10.3390/pathogens14030225

**Published:** 2025-02-25

**Authors:** Geovana Thaís Motta, Aline Fernanda dos Santos, Paulo Henrique Campos, Flavio Luiz de Oliveira, Margarete Schinemann, Nariany Pollyane da Silva, Pricila Regina Sikora Bruger, Kauane Oliveira Campos, Luciana Dalazen dos Santos, Carla Fredrichsen Moya, Meire Christina Seki, Adriano de Oliveira Torres Carrasco

**Affiliations:** 1 Graduate Program in Veterinary Sciences, State University of the Midwest of Paraná (UNICENTRO), Guarapuava 85040-167, PR, Brazil; geothmotta@hotmail.com (G.T.M.); paulohcampos2000@gmail.com (P.H.C.); 2 Laboratory of Infectious and Parasitic Diseases (LADIP), State University of the Midwest of Paraná (UNICENTRO), Guarapuava 85040-167, PR, Brazil; aline.santos@outlook.com.br (A.F.d.S.); lucianadalazen@gmail.com (L.D.d.S.); carlafredrichsen@yahoo.com.br (C.F.M.); meireseki@hotmail.com (M.C.S.); 3 Graduate Program in Animal Science, Federal University of Paraná, UFPR, Palotina 85950-000, PR, Brazil; 4 Municipal Department of Agriculture and Livestock of the Municipality of Turvo, Turvo 85150-000, PR, Brazil; 5 Municipal Health Department of Turvo, Turvo 85150-000, PR, Brazil

**Keywords:** feline, public health, sporothrix, zoonosis

## Abstract

This study aims to report the number of animal cases identified in the central-south region of Paraná. It also seeks to correlate these findings with human diagnoses, thereby underscoring the importance of the One Health approach in implementing prophylactic measures and protocols for evaluating both positive and suspected cases. In August 2023, a cat diagnosed with sporotrichosis was presented at the UNICENTRO Veterinary School Clinic. Accompanying the cat was its guardian, who exhibited characteristic lesions of the disease. An epidemiological study was then initiated and spanned from August 2023 to March 2024. Cytological tests were performed on the suspected cases. From the epidemiological survey, 21 animals were tested. A total of 15 cats were confirmed to have sporotrichosis; all were unneutered males of the moggy breed with access to outdoor environments. Some pet owners also displayed symptoms and lesions consistent with sporotrichosis. Considering the recent surge in sporotrichosis cases in Brazil, along with its zoonotic potential and significance for public health—and considering its status as a notifiable disease—epidemiological studies such as this one are vital. They help in understanding the spread of the disease and are crucial for the development and implementation of prophylactic measures to protect human and animal health.

## 1. Introduction

Although zoonosis remains a major global concern, developing countries remain at higher risk of zoonotic diseases, given the nature of contact between animals and humans, limited surveillance capacities and the limited availability of resources [1]. Sporotrichosis is a fungal infection that impacts both humans and animals, particularly cats, and is prevalent primarily in tropical and subtropical regions. It is considered endemic in Latin America [2,3]. In cats, sporotrichosis results in the formation of papular-nodular lesions during the advanced clinical phase or ulcerative lesions in the later stages [4]. The disease was first documented in the United States in 1898 by Benjamin Schenck, who isolated the fungus from a lesion on a human’s upper limb [5]. The first case in Brazil was described by Freitas et al. (1956) in Minas Gerais, involving a feline with extensive skin lesions [6]. The causative agent of sporotrichosis is the fungus from the genus *Sporothrix*, which is part of the Ophiostomataceae family and exhibits dimorphism. This means it has two distinct forms, depending on the growth temperature of the agent: a filamentous and a yeast form. Species within this genus include *S. schenckii*, *S. brasiliensis*, *S. globosa*, *S. mexicana*, *S. luriae* and *S. albicans*. Studies indicate that *S. schenckii* and *S. brasiliensis* are the primary species infecting humans and cats in Brazil. In the environment, the fungus is commonly found on rose thorns and is saprophytic in soil and vegetation, often referred to as the “rose gardener’s disease” [4,7,8].

Felines are the principal hosts and vectors of the disease, which most frequently affects adult male cats of mixed breeds that are not neutered and have access to contaminated environments. Transmission can occur through direct contact with the fungus in nature, via contaminated soil or plant material, through enzootic horizontal transmission (cat-to-cat/dog-to-cat), and zoonotic horizontal transmission (cat-to-human) via scratches, bites or contact with the lesions of an infected cat. Occasionally, the disease can also spread through alternative routes such as airborne or digestive pathways, leading to systemic infections [4,9,10,11]. Given its significance for both animal and human health, it is necessary to adopt a range of strategies to manage the disease. These include accurate diagnosis, isolation and treatment of infected cats; control of stray animal populations; and public health education on responsible pet ownership. To reduce environmental contamination, it is crucial to incinerate deceased infected animals rather than burying them, to prevent soil contamination [10].

Anamnesis and physical examination are crucial tools for diagnosing diseases in both humans and animals. It is important to gather information on the progression and location of the lesion, previous therapies employed, immunosuppressive conditions, exposure to potential infection sources, access to the outdoors and contact with other animals. A detailed analysis of the lesions should also be performed. However, laboratory tests are necessary for a definitive diagnosis [10,11].

During the physical examination, the cutaneous form of the disease in cats typically presents as ulcerated, nodular, granulomatous and pyogranulomatous lesions, predominantly affecting the face and limbs. In cases of extra-cutaneous sporotrichosis, there is a risk of dissemination to the lymphatic, respiratory and neurological systems, potentially leading to conditions such as lymphadenopathy, sinusitis, rhinosinusitis, pneumonia and meningoencephalitis [12,13].

Various techniques are available for diagnosing sporotrichosis. The gold standard for identifying *Sporothrix* spp. is fungal culture, which produces creamy, clear colonies with an irregular appearance, although this method is more costly. Cytological examination through imprint or fine needle aspiration cytology (FNAC) is another option, revealing small, ovoid, spherical, cigar-shaped leviduriform structures, typically surrounded by a clear, thin halo in the cytoplasm. However, this method has limitations and may yield false-negative results. Serological tests, known for their high sensitivity and specificity, are utilized alongside the clinical monitoring of infected animals until the infection is completely resolved [10,14,15,16,17].

In recent decades, there has been a significant increase in our understanding of sporotrichosis among animals and humans in Brazil [18]. According to data from the Oswaldo Cruz Foundation, 4188 human cases were identified in Rio de Janeiro in 2011, and by 2015, the number of diagnosed cats had risen to 4703. Although underreported, the incidence of sporotrichosis in cats is rising across all Brazilian regions, reaching the status of a national epidemic [19].

In the state of Paraná, both human and animal cases of sporotrichosis are subject to mandatory reporting. In 2022, 1319 cases in animals were documented [20]. In response, the state issued Joint Technical Note No. 6/2023, announcing the provision of free medications for animals diagnosed with the disease as part of the unified health system [21].

Given the significant impact of sporotrichosis on both animal and human health, this study aims to report the number of animal cases identified in the central-south region of Paraná from 1 August 2023, to 31 March 2024. It also seeks to correlate these findings with human diagnoses, thereby underscoring the importance of the One Health approach in implementing prophylactic measures and protocols for evaluating both positive and suspected cases.

## 2. Materials and Methods

**Figure 1:**
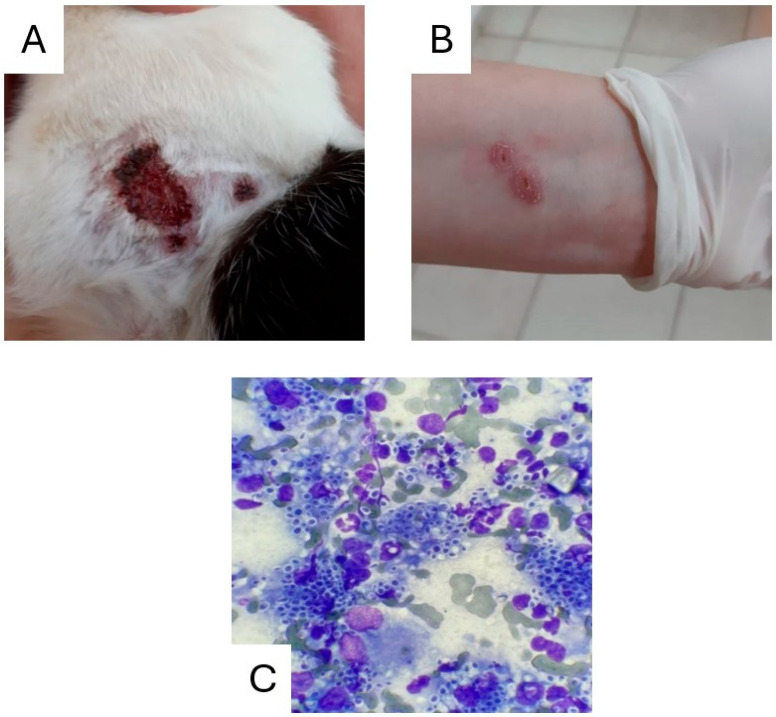
(**A**) Ulcerative skin lesion in a feline treated at the Veterinary School Clinic, CEVET. (**B**) Papulo-nodular skin lesion on the animal’s guardian. (**C**) Cytology carried out by imprinting with rapid panoptic staining, with rounded and cigar-shaped structures compatible with sporotrichosis.

In August 2023, a cat from a city in the central-south Region of Paraná was treated at the Veterinary School Clinic (CEVET) of the State University of Centro-Oeste (UNICENTRO) in Guarapuava-PR for a lesion on its right thoracic limb (Figure 1A). During the anamnesis, the cat’s guardian described a persistent lesion on his left arm (Figure 1B) that had not healed, despite antibiotic treatment. After examining the cat’s ulcerative lesions and taking samples for imprint cytology, the slides were sent to the Laboratory of Infectious and Parasitic Diseases (LADIP) at the same university and processed using the rapid panoptic staining technique.

Following this, the case was reported to the Ministry of Health’s SINAN system (Information System for Notifiable Diseases). Subsequently, an Epidemiological Survey was conducted in the municipality by the Health Department from 1 August 2023 to 31 March 2024. This survey involved taking samples from 21 unneutered cats with free street access, all exhibiting lesions compatible with sporotrichosis, and included clinical assessments of the humans living with these animals. Evaluated animals were referred due to the presence of clinical signs compatible with sporotrichosis. All animal procedures were performed according to the Ethical Principles in Animal Research adopted by the Brazilian Society for Laboratory Animal Science and to the 2020 Report of the AVMA Panel on Euthanasia [22].

In this epidemiological assessment, directed at populations with the presence of animals or people exhibiting lesions compatible with sporotrichosis, fine needle aspiration cytology (FNAC) and imprinting techniques were employed on the lesions. The samples were then sent to the Laboratory of Infectious and Parasitic Diseases (LADIP), where the slides were stained using the rapid panoptic technique and examined under light microscopy at 40× and 100× magnifications. Samples from the lesions of the individuals responsible for the animals were also sent to a reference laboratory for diagnosis by the Municipal Health Department. People had a history of wounds on the skin and mucous membranes of the eyes, nose and mouth, with a history of lack of response to antibiotic treatment.

## 3. Results

Clinical evaluations of the animals and the sample collection were performed by resident veterinary doctors specializing in infectious and parasitic diseases. The physical examinations revealed skin lesions characterized by inflammation, ulceration and scabbing, primarily on the face and, in some cases, the limbs. Samples obtained from the first animal evaluated revealed rounded and cigar-shaped structures under optical microscopy at 40× and 100× magnification, confirming the suspicion of sporotrichosis, as illustrated in Figure 1C.

**Figure 2:**
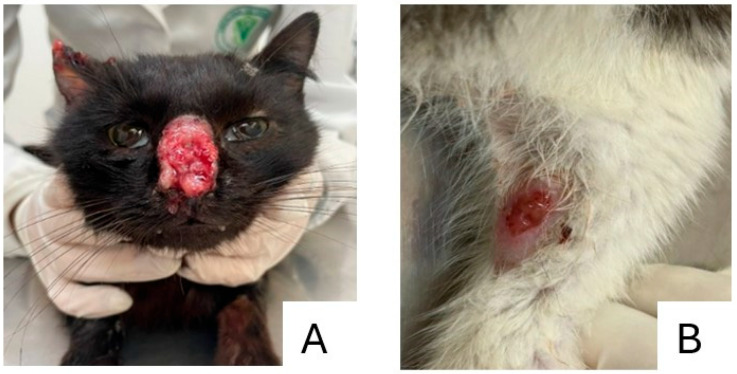
Representation of two cats with lesions compatible with sporotrichosis, treated at the Veterinary School Clinic—CEVET. (**A**) Skin lesion on the right nostril and ear. (**B**) Skin lesion on the right pelvic limb.

**Figure 3:**
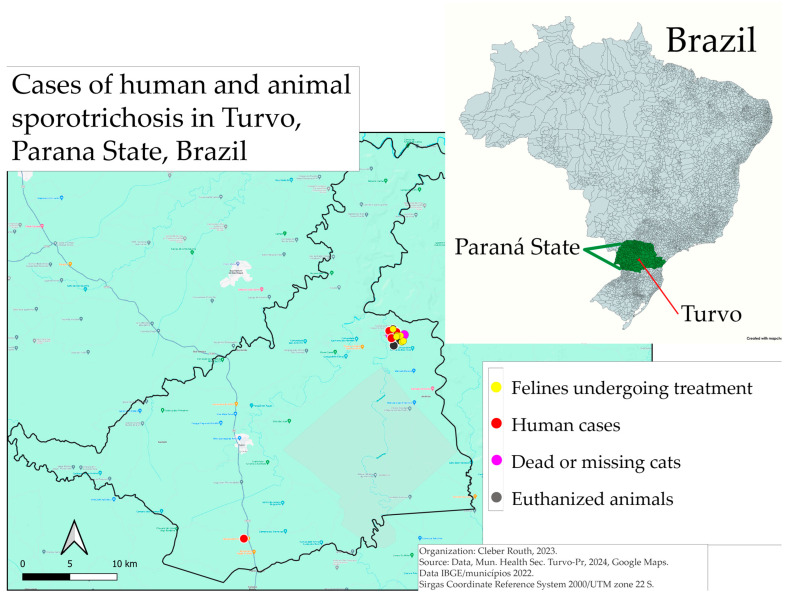
Localization of cases in the municipality of Turvo, Parana State, Brazil, listing cases in cats, humans, dead/missing animals or euthanized animals.

From the epidemiological survey conducted, 21 animals were tested—2 females and 19 males; all were moggy, unneutered and had street access. Of these, 15 male cats tested positive for sporotrichosis. It was also noted that at least one person in the households responsible for these animals exhibited symptoms and lesions consistent with sporotrichosis (Figure 2), including persistent sores that showed no improvement and were unresponsive to antibiotic treatment. A total of four humans have been diagnosed and are under treatment. These people were suspected of having the agent due to the occurrence of lesions associated with contact with cats. The locations of the positive samples from humans and animals, along with details of animals that either died from other causes or are missing, are depicted in Figure 3. This part of the municipality characterized by vulnerable living conditions. Given the occurrence of these cases in the municipality, the health team received training to recognize clinical suspicions, collect samples and guide the population.

After confirming the positive diagnoses of sporotrichosis, treatment was initiated for animals until the definitive remission of symptoms, verified by serological testing. The treatment period with Itraconazol lasted no less than four months. Some animals were euthanized due to conditions deemed incompatible with life, according the AVMA Panel on Euthanasia [22]. The diagnosis of sporotrichosis was confirmed in four more people: two adults and two children. After the diagnosis of human cases, treatment with antifungals (Itraconazol) was established and there was a complete remission of the condition.

## 4. Discussion

The saprophytic fungus *Sporothrix* spp. favors warm and humid climates for its development [20,23]. Although the central-south region of Paraná, located in southern Brazil, has a moderate subtropical climate with an annual average temperature of 16.8 °C, which typically does not promote the spread of this fungus, there has been a notable increase in cases. According to preliminary data from the Notifiable Diseases Information System (SINAN) [20], only five cases were recorded from January to July 2023. This figure may be an underestimate due to underdiagnosis of the disease in both humans and animals, as the region had not previously focused significantly on this disease. Two essential steps in surveillance of diseases at a local or national level are complete and fast detection and the reporting of incidents of communicable diseases [24,25].

Supporting these observations, Santos et al. [26] noted that male, fertile cats are more susceptible to the disease, likely due to territorial conflicts; thus, castration is an effective control measure. Additionally, promoting responsible pet ownership and discouraging animal abandonment positively impacts the control of infectious diseases and should be incorporated into each community’s health service awareness programs [27,28].

In the study by Poester et al. [29] in Rio Grande do Sul, the cats that tested positive for sporotrichosis were predominantly male, moggy and had outdoor access, consistent with the epidemiological findings. However, at least half of these animals were neutered, unlike all the cases in this study. Similarly, Chaves et al. [30] in Rio de Janeiro found that most of the cats studied were male and moggy, but more than half were neutered. Madrid et al. [31] reported 15 cases of feline sporotrichosis in the region of Pelotas, Rio Grande do Sul, where all animals had free access to the streets and were unneutered, mirroring the cases in the central-south region of Paraná.

The area where these animals and people were diagnosed is a part of the municipality characterized by vulnerable living conditions, corroborating findings by Chaves et al. [30], who noted that the majority of the studied population lived with low economic status. Figure 3 illustrates that the occurrence of cases in humans and animals is concentrated in a specific region of the municipality. Regarding the human case in a more remote and equally vulnerable area, it was not possible to determine the site of infection due to the long progression of the lesion over more than eight months, with the infected individual unable to recall the time and place of potential contact with the cat. It should be noted that some animals either died from other causes or disappeared, as they are feral. Barros et al. [32] state that most sporotrichosis cases occur in peripheral urban areas characterized by low incomes, which reflects a socioeconomic vulnerability. Furthermore, Gremião et al. [12] suggest that the spread of sporotrichosis may be linked to poverty, inadequate sanitation and human environmental alterations, emphasizing the social vulnerabilities faced by the population. The prevention of sporotrichosis represents another urgent need for public health preparedness, readiness and response through the human–animal–environment interface using the One Health (OH) approach [1].

Given that sporotrichosis is considered an emerging fungal infection, it is essential to collect, process and interpret epidemiological data to define various control and prevention strategies aimed at reducing public health risks associated with zoonotic outbreaks, highlighting the crucial role of veterinarians in One Health [31,32,33]. This role was pivotal in the described case, as human cases were only identified following the veterinary evaluation of a cat. From this initial diagnosis, it was possible to detect the agent in many other animals and humans.

The adoption of health education actions applied to the most affected population was positive for preventing and identifying new cases (animals and humans). Training was carried out with the local health team to enable the faster identification of new cases. Educational actions were also carried out with the population, aimed at children and adults, so that they could seek a diagnosis for their animals if they showed any signs of the disease. Disease surveillance indices must be routinely measured and improved at both governmental and private sectors. Training health personnel, especially physicians, about the objectives of the surveillance system and its importance on a periodic base is necessary [24]. Health teams—including doctors, veterinarians, health workers and other professionals—must remain vigilant regarding the emergence of illnesses. An integrated notification system is essential to accelerate diagnoses and implement targeted prophylactic measures.

## 5. Conclusions

Given the recent surge in cases of the disease in Brazil, coupled with its zoonotic nature, public health significance and mandatory reporting requirements, epidemiological studies like this one are crucial for both human and animal health. Such studies enable the implementation of effective prophylactic measures. It is essential for all members of health teams—including doctors, veterinarians, nurses and other health professionals—to be well-informed about the clinical signs of the disease to ensure accurate treatment and control strategies.

## Data Availability

https://www.saude.pr.gov.br/Pagina/Zoonoses (accessed on 18 February 2025).

## Abbreviations

The following abbreviations are used in this manuscript:

FNAC	Fine needle aspiration cytology
CEVET	Veterinary School Clinic
LADIP	Laboratory of Infectious and Parasitic Diseases
SINAN	Information System for Notifiable Diseases
AVMA	American Veterinary Medical Association
OH	One Health
